# Cognitive Factors Influencing COVID-19 Vaccination Intentions: An Application of the Protection Motivation Theory Using a Probability Community Sample

**DOI:** 10.3390/vaccines9101170

**Published:** 2021-10-12

**Authors:** Kwok Kit Tong, Mu He, Anise M. S. Wu, Le Dang, Juliet Honglei Chen

**Affiliations:** 1 Department of Psychology, Faculty of Social Sciences, University of Macau, Macao, China; kktong@um.edu.mo (K.K.T.); yc07309@um.edu.mo (M.H.); anisewu@um.edu.mo (A.M.S.W.); yb97304@um.edu.mo (L.D.); 2 Centre for Cognitive and Brain Sciences, Institute of Collaborative Innovation, University of Macau, Macao, China; 3 Faculty of Teacher Education, Pingdingshan University, Pingdingshan 467000, China

**Keywords:** COVID-19, protection motivation theory, intention, vaccine efficacy, vaccine acceptance

## Abstract

COVID-19 vaccines have been shown to provide protection against severe disease and death. However, substantial individual differences in vaccination intentions have hindered achieving optimal vaccination rates across the population. To look for efficient strategies to promote vaccination, this study tested whether the protection motivation theory (PMT), a cognitive model based upon threat and coping appraisals, would account for the differences in vaccination intentions under three scenarios (i.e., in the context of getting vaccinated in general, and in the context of high- and low- efficacy for reducing COVID-19 transmission risk). A phone survey was conducted in early 2021 and obtained a probability community sample (*n* = 472; 49.2% men) in Macao, China. We found that 54.0% of respondents indicated their relatively strong intention to receive COVID-19 vaccination for high-efficacy vaccines, compared to 29.5% for low-efficacy vaccines and 31.0% for vaccines in general. After adjusting for demographics, self-efficacy (i.e., the perceived capability of receiving COVID-19 vaccines) and maladaptive response reward (i.e., the perceived benefits of not receiving COVID-19 vaccines) were consistently associated with COVID-19 vaccination intentions under all three scenarios. The perceived severity of COVID-19 infection and response cost (i.e., the perceived costs of receiving COVID-19 vaccines) were significantly associated with vaccination intention for high-efficacy vaccines, while the response efficacy of lowering the COVID-19 impact with COVID-19 vaccination was positively associated with vaccination intention for general and low-efficacy vaccines. Given that the relative strength of PMT constructs depends on perceived vaccine efficacy, we recommend taking PMT constructs and vaccine efficacy into account for encouraging vaccination.

## 1. Introduction

As of 20 May 2021, the infectious disease COVID-19, caused by SARS-CoV-2, had led to over 166.3 million confirmed cases and over 3.4 million deaths [1]. Since the end of December 2020, the World Health Organization (WHO) had identified several COVID-19 vaccines that have been shown to be efficacious in lowering the risk of contracting COVID-19 during controlled clinical trials in the WHO Emergency Use Listing [2]. As crucial tools in the pandemic response, COVID-19 vaccines also demonstrated accumulating evidence of protection against severe disease, hospitalization, and death [3,4,5]. While vaccination provides individuals protection against the virus [6], researchers also advocate a high-level community vaccination coverage to minimize the impact of COVID-19 on society [7]. Unfortunately, past studies have indicated that vaccine hesitancy might hinder one’s intention to get vaccinated. For example, studies on the H1N1 pandemic influenza showed that the vaccine uptake rate was well below 50% despite its severity [8]. The indecisiveness about or refusal of specific vaccines or vaccination in general may be attributed to contextual influences (e.g., facilities and media coverage), individual and group influences (e.g., perceived risk, benefit, and norms), and vaccine-specific issues (e.g., the strength of recommendation) [9]. Understanding and improving individuals’ intentions to receive vaccination has always been a research focus, especially for non-mandatory vaccinations such as for influenza and HPV [10,11]. There were substantial individual differences in vaccine acceptance, and factors that account for the differences can be context-specific and vary across vaccines [12]. Concerning COVID-19, Anderson et al. [13] argued that encouraging COVID-19 vaccination to achieve a high rate in the population would be challenging because of vaccine hesitancy. SARS-CoV-2, the virus that induced COVID-19, may become endemic if a high vaccine coverage cannot be reached, and vaccine delay or refusal may make global COVID-19 responses less effective [14].

Based on the protection motivation theory (PMT), the present study aimed at identifying the cognitive factors underlying individual differences in intentions towards COVID-19 vaccination. In PMT, protection motivation, the intention to perform a health behavior (e.g., vaccination), is influenced by six health cognitions through a threat appraisal process (i.e., evaluating factors that increase or decrease the probability of maladaptive responses) and a coping appraisal process (i.e., evaluating factors that increase or decrease the probability of adaptive responses to deal with a threat) [15]. The six health cognitions are perceived severity (i.e., how serious one perceives the consequences of a health threat), perceived vulnerability (i.e., how likely one perceives his/her exposure to the threat), maladaptive response reward (i.e., how beneficial one perceives the performing of maladaptive responses), response efficacy (i.e., how effective one perceives the adaptive response in dealing with the health threat), self-efficacy (i.e., how capable one perceives him/herself to perform the protective behaviors), and response cost (i.e., how much cost one perceives might arise from acting on the protective behaviors) [15]. The former three health cognitions are involved in the threat appraisal process, while the latter three are more likely to be considered during the coping appraisal process. These six health cognitions (hereinafter *PMT variables*) have been utilized to predict health-related intentions and behaviors (e.g., cancer and AIDS prevention, healthy lifestyle, and medical adherence), with a moderate effect size [16], indicating its efficacy in developing health interventions. Bish et al. [17] suggested that PMT is a useful framework for understanding the cognitive factors underlying vaccination decisions. Subsequent empirical studies have substantiated the predictive effect of the PMT variables on the intention to receive vaccination [18,19]. Other researchers also used PMT variables to explain vaccination decisions during the H1N1 pandemic, demonstrating the usefulness of PMT in informing effective interventions on promoting vaccination uptake [20]. However, there were inconsistencies in the findings concerning the relevancy of different PMT variables. For instance, Camerini et al. [21] found that only response efficacy predicted the vaccination of measles, mumps, and rubella (MMR); in contrast, Liu et al. [22] showed that perceived severity, self-efficacy, and response cost, but not response efficacy, predicted MMR vaccination.

As for COVID-19 vaccination intention, there is limited and inconclusive evidence concerning the effectiveness of PMT variables. An online survey of university students in China showed that only the perceived severity of the disease, but not the other PMT variables, significantly explained positive vaccination intentions [23]. On the contrary, in another study based on a snowballing adult sample and single-item measurements, coping appraisal variables (e.g., response efficacy and response cost), but not threat appraisal variables (e.g., perceived severity), were associated with COVID-19 vaccination intention [24]. These inconsistent findings may be understandable in light of an argument that the role of PMT variables is specific to the behaviors across target groups and situations (e.g., at a different stage of the pandemic and with/without information about vaccines) [16,25]. Nevertheless, the existing literature provides insufficient information regarding the roles of various PMT variables on COVID-19 vaccination intentions, which is an essential piece of information to devise an effective plan to boost vaccination rates to achieve a high level of community vaccination coverage. In addition, while there may be differences in the efficacy between vaccines [26] and between individuals [27], the efficacy of a vaccine may influence the relative predictive strengths of the PMT factors for vaccination intentions [28]. In our study, on top of the general intention regarding COVID-19 vaccination, we also measured people’s intention to receive high- (i.e., 95%) and low- (i.e., 50%) efficacy vaccines in order to explore whether different salient factors would emerge. Such findings could provide practical implications in designing promotion strategies for countries where different vaccines are available.

In summary, the present study aimed at testing the relation between the PMT variables and COVID-19 vaccination intentions using a probability community sample. We hypothesized that two threat appraisal constructs (i.e., perceived severity of the disease and perceived vulnerability to the disease) and two coping appraisal constructs (i.e., response efficacy [in terms of one’s perceived cost of performing the protective behavior] and self-efficacy [in terms of one’s perceived capability of performing the protective behavior]) would be positively associated with vaccination intention; on the contrary, maladaptive response reward from the threat appraisal and response cost from the coping appraisal would be negatively associated with vaccination intention. Additionally, this study also explored whether the explanatory power for the PMT variables would vary across scenarios when different vaccine efficacy levels are considered.

## 2. Materials and Methods

### 2.1. Respondents and Procedure

A telephone survey (see Appendix A for detailed items) was conducted by our research team from January to February 2021 to obtain a probability community sample in Macao, China. A random sample of households was first selected from the latest residential phonebook with a computerized phone poll system. Within each household, we applied the last birthday rule [29] to perform subsequent random selection if multiple eligible residents met our inclusion criteria (i.e., local adult residents who could speak Cantonese or Mandarin Chinese). Participation was anonymous, voluntary, and without monetary incentives. All chosen respondents received a briefing on the study information and participation rights. Only those who gave verbal consent to participate in the study proceeded to the formal interview process. To ensure data integrity, we offered intensive training to all the interviewers on how to properly conduct standardized interviews and only hired those who passed the final evaluation to perform formal data collection (passing rate = 93.9%). Furthermore, a group of experienced project administrators was hired to oversee the whole data collection process and perform regular data quality checks.

Using GPower 3.1 [30], we identified 342 as the minimum sample size with an 80% power [31] to detect a small effect size (i.e., *f*^2^ = 0.047; [32]) for our desirable statistical analyses at the significance level of 0.05. Eventually, we obtained a probability sample of 472 Chinese adults (49.2% men). The cooperation rate was 83.9%, following the calculation method proposed by the American Association for Public Opinion Research [33]. The average age of the respondents was 40.28 years (*SD* = 13.67; *range* = 18 to 81). The majority of the respondents had received education at the college level or higher (59.3%).

### 2.2. Measures

#### 2.2.1. COVID-19 Vaccination Intentions

During the survey period, the local government had announced their rollout plan of COVID-19 vaccines for all adult residents, which included three types of vaccines (i.e., an mRNA vaccine, an inactivated whole virus vaccine, and a replication-defective adenovirus vector vaccine); however, the vaccination rollout had not yet started during the survey period [34]. Because there were no widely accepted efficacy figures for each vaccine, we used three items to measure the COVID-19 vaccination intention under three conditions. First, we asked respondents to indicate the intensity of their intention to receive a COVID-19 vaccine without mentioning any information regarding the vaccine efficacy (hereinafter *general vaccination intention*). Subsequently, we also asked respondents their intention to receive the vaccination when its efficacy against COVID-19 was 50% (i.e., those who received the vaccine were at a 50% lower risk of contracting COVID-19 than the group who received the placebo based on the results of controlled clinical trials; hereinafter *low-efficacy vaccine intention*) and 95% (i.e., those who received the vaccine were at a 95% lower risk of contracting COVID-19 than the group who received the placebo based on the results of controlled clinical trials; hereinafter *high-efficacy vaccine intention*), respectively. A 5-point Likert response scale (1 = *very low*, 5 = *very high*) was used to measure the extent of one’s vaccination intention, and a higher score represented a stronger intention.

#### 2.2.2. PMT Variables

Items of the six PMT variables were either adapted from previous studies or constructed based on the current study’s context. All the items adopted a 5-point Likert response scale (1 = *strongly disagree* to 5 = *strongly agree*), with a higher score indicating a higher level of agreement. A small pilot sample (*n* = 10) tested these items and generally agreed that all items could be easily comprehended. The six PMT variables contained three threat appraisal variables and three coping appraisal variables. The three threat appraisal variables are (a) perceived severity of COVID-19 (hereinafter *perceived severity*), which included three items adapted from Tong et al. [32], with a sample item being, “If you have COVID-19, your study or career will be harmed” (*α* = 0.69); (b) perceived vulnerability to COVID-19 (hereinafter *perceived vulnerability*), also adapted from Tong et al. [32], containing three items (e.g., “You feel there is a high chance for you to contract COVID-19”) and a Cronbach’s alpha of 0.69; and (c) the three-item scale of maladaptive response reward of not receiving COVID-19 vaccines (hereinafter *maladaptive response reward*; e.g., “Not receiving COVID-19 vaccines can save you from troubles.”), with a Cronbach’s alpha of 0.83. The three coping appraisal variables are (a) self-efficacy of receiving COVID-19 vaccines (hereinafter *self-efficacy*), which followed the suggestion of Hoeppner et al. [35] and was measured with a single item (i.e., “You believe that you are able (e.g., having the time and resources) to receive COVID-19 vaccines.”); (b) response efficacy of vaccinations (hereinafter *response efficacy*), assessed one’s perceived effectiveness of vaccination in reducing the COVID-19 risk [21] with two items (e.g., “Receiving COVID-19 vaccines can lower your risk of contracting COVID-19”) and showed a reliability of 0.89; and (c) response cost of vaccination (hereinafter *response cost*), assessed by three items regarding the perceived barriers to and side effects from vaccination [21,22], with a sample item being “You may experience side effects due to COVID-19 vaccination” and with an internal consistency of 0.76.

#### 2.2.3. COVID-19 Related Experiences

COVID-19 related experiences were collected from all the respondents, including being diagnosed with COVID-19, close contact with confirmed COVID-19 cases, and quarantine because of the COVID-19 pandemic. Each respondent was asked about whether they, their family members, or friends had any of such experiences.

#### 2.2.4. Demographic Variables

Respondents’ demographic information was collected, including sex, age, and educational attainment.

### 2.3. Data Analysis

SPSS 26 was used to conduct data analysis, including descriptive statistics, *t*-test, *F*-test, bivariate correlation, and linear regression.

## 3. Results

### 3.1. Descriptive Statistics

Among the overall 472 respondents, there were 232 men (49.2%; 95% CI [44.7%, 53.7%]) and 240 women (50.8%; 95% CI [46.3%, 55.3%]). Their average age was 40.28 years (*SD* = 13.67; *range* = 18 to 81). The age group distribution of our respondents was 8.4% of 18–24 years, 30.8% of 25–34 years, 32.6% of 35–44 years, 12.0% of 45–54 years, 7.8% of 55–64 years, and 8.4% of 65 years and above. Around sixty percent of the respondents had received education at the college level or higher, whereas 34.9% had received secondary schooling and 5.8% were at the primary school level or lower. Regarding COVID-19 related personal experience, none of the 472 respondents had been diagnosed with COVID-19, one had close contact with confirmed COVID-19 patients, and six had been quarantined. Furthermore, five respondents indicated that their family members had experienced quarantine, but none of them had been diagnosed with COVID-19. Two respondents indicated that their friends had been diagnosed with COVID-19, and six indicated that their friends had had a quarantine experience.

Given the 5-point Likert response scale used for measuring vaccination intentions, we adopted the middle point (i.e., 3 = *a medium level*) as the reference point and categorized those respondents who endorsed 4 = *strong* or 5 = *very strong* as “strong intention” and those who scored 2 = *low* or 1 = *very low* as “weak intention”. For the general vaccination intention (*M* = 3.06, *SD* = 1.02), 31.0% of respondents belonged to the strong intention towards vaccination group, while 26.5% were members of the weak intention towards vaccination group. For the intention to receive the low-efficacy vaccines (*M* = 3.00, *SD* = 1.04), 29.5% were of strong intention and 29.1% were of weak intention. In contrast, the intention to receive high-efficacy vaccine (*M* = 3.55; *SD* = 1.12) showed 54.0% with a strong intention and 18.0% with weak intention. A paired-sample *t*-test showed that respondents’ intentions towards high-efficacy vaccines were significantly higher than that towards low-efficacy vaccines (*t* (462) = 13.05, *p* < 0.001), and this preference for high-efficacy vaccines was consistent across both sexes, all age groups, and all education attainment groups (see Table 1, “Low- vs. high- efficacy (*t*-test)”; *t* = −10.39 to −4.17, *p* < 0.001).

To test the effect of demographic variables on vaccination intentions, a series of independent *t*-tests was conducted to compare the mean difference in intention to receive different types of vaccines between men and women, while an analysis of variance (ANOVA; also known as *F*-test) was conducted among the respondents of all age groups and educational attainment groups to evaluate the potential age and educational differences in intention for each type of vaccine. As shown in Table 1, women had a significantly higher intention to receive the high-efficacy vaccines than men. Respondents of different age groups had significantly different intentions to receive the vaccines in the general and high-efficacy scenarios. Specifically, people who were 55 to 64 years old had the highest general vaccination intention, and their intention was significantly higher than people who were 25 to 44 years old. People who were 25 to 34 years old had the lowest general vaccination intention, which was significantly lower than people younger than 24 years old or older than 55 years old. Regarding high-efficacy vaccines, people who were 35 to 44 years old had the lowest vaccination intention, which was significantly lower than people younger than 24 years old or older than 45 years old. In addition, we did not observe a significant educational difference for vaccination intention under the general nor high-efficacy scenario; however, people with secondary school educational attainment reported a significantly higher intention to receive low-efficacy vaccines than people from other educational attainment groups.

Table 2 shows the bivariate correlations among vaccination intentions and PMT variables. As hypothesized, self-efficacy and response efficacy were both positively correlated to three types of intention (*r* = 0.27 to 0.43, *p* < 0.001), while maladaptive response reward and response cost were negatively related to three types of intention (*r* = −0.20 to −0.46, *p* < 0.001). However, perceived severity was only significantly correlated with high-efficacy vaccine intention (*r* = 0.30, *p* < 0.001).

### 3.2. Regression Analysis

The explanatory value of each PMT variable was further explored with hierarchical regression analysis, in which sex, age, and educational attainment were controlled for (see Table 3). Maladaptive response reward (*B* = −0.30 to −0.33, *p* < 0.001) and self-efficacy (*B* = 0.16 to 0.24, *p* < 0.001) were significantly associated with all three types of intention. Response efficacy was significantly and positively associated with vaccination intention under both general and low-efficacy scenarios (*B* = 0.29 and 0.28, respectively, *p* < 0.001). For high-efficacy vaccines, vaccination intention was also associated with perceived severity (*B* = 0.30, *p* < 0.001), and response cost (*B* = −0.17, *p* < 0.05).

## 4. Discussion

Our findings showed that the general vaccination intention was low, with fewer than one-third of the respondents having a strong intention. In contrast, the high-efficacy vaccination intention was relatively high, with more than half of the respondents displaying a strong intention. Consistent with previous studies, e.g., [36], these results indicated that vaccine efficacy was an important determinant for reducing vaccine delay or refusal. From a practical perspective, promotional materials could emphasize vaccine efficacy as a strategy to enhance the willingness for COVID-19 vaccine uptake and accelerate the pace of achieving a high rate of community vaccination coverage. Admittedly, one shall also take into account the mistrust and fear of side effects from COVID-19 vaccines to better understand the motivation behind not getting vaccinated, for a discussion see [37]. Aside from clarifying misinformation against vaccines [38], it may also be helpful to provide residents with more information on why a particular type of vaccine can be effective (e.g., its underlying mechanisms). In addition, in line with past findings [39,40], we observed a significant age difference between respondents’ intention for vaccination under both general and high-efficacy scenarios—older people, especially in the 55–64 years-old group, presented a stronger intention than their relatively younger counterparts. The other demographic effects, such as sex and educational attainment, appeared to be less consistent for accounting for the differences in vaccination intentions across the subgroups; nevertheless, every demographic subgroup consistently indicated a significantly stronger intention to receive high-efficacy vaccines than low-efficacy ones regardless of one’s sex, age, and educational attainment. We inferred that enhancing the perceived efficacy of the vaccines could be a cost-effective promotion strategy for the public in general through approaches such as reducing misinformation (i.e., countering the misconceptions regarding vaccine efficacy).

The present study provided support for the applicability of PMT to explain individual differences in COVID-19 vaccination intentions in the context of beliefs about vaccine efficacy for lowering COVID-19 risk. Our findings were generally consistent with the mainstream findings reported in the past studies regarding vaccination intention [18,19,22]. In our study, self-efficacy (i.e., perceived capability to get vaccinated) and maladaptive response reward (i.e., perceived benefits of not getting vaccinated) explained the variances in intentions to receive different types of COVID-19 vaccines. While some studies argued that the relative strength of variables might vary across samples and situations [16,25], our findings further hinted at the possibility that the relative strength of the PMT variables may vary for different vaccines, even for the same disease. We showed that for high-efficacy COVID-19 vaccines, the perceived severity of COVID-19, the self-efficacy of getting vaccinated, and the maladaptive response reward of not receiving vaccines were significant factors for vaccination intentions. This pattern was similar to the findings for MMR vaccination intention [22]. In contrast, response efficacy, along with self-efficacy and maladaptive response reward, emerged as salient factors of vaccination intention for low-efficacy vaccines. The differences between intentions towards vaccines of high- and low- efficacy levels suggested that careful considerations should be given to the generalization and comparison of findings concerning COVID-19 vaccine acceptance across regions/countries with vaccines of different efficacy. In places where a relatively low-efficacy vaccine is adopted, the promotion strategy may need to focus on response efficacy.

Many studies applying PMT on vaccination intentions have shown that either coping appraisal variables (e.g., response efficacy) or threat appraisal variables (e.g., perceived severity and perceived vulnerability) could explain the variances in vaccination intention, but not both [21,22,23,24]. However, our findings, consistent with past studies [41], suggested that the PMT variables from both threat appraisal and coping appraisal contributed to the understanding of COVID-19 vaccination intentions, providing additional support for applying PMT to the promotion of vaccination. Nevertheless, we took a closer look into the extant COVID-19 vaccination studies that reported a line of findings different from ours, e.g., [23,24], and speculated that these variations might be related to the sample under study or the vaccine being adopted for the samples. Some researchers have also pointed out that the applicability of PMT may be sensitive to sample characteristics or situations, e.g., [16,25], such as vaccine efficacy, perceived vaccine safety, and concerns over the possible side effects. Given that it is not uncommon that vaccines for the same disease may have different efficacies [42], the present study highlighted the importance of considering the efficacy of vaccines being adopted within a country or a region to devise a better promotion plan to overcome vaccine hesitancy.

At a practical level, we may want to focus more on factors that may hinder vaccination intention when the vaccine efficacy is low. Based on our findings, we believe that interventions for enhancing self-efficacy and altering maladaptive response reward would effectively encourage COVID-19 vaccination even if the vaccine’s efficacy is low. Based on Bandura’s self-efficacy theory [43], self-efficacy enhancement can be commonly achieved by equipping people with the knowledge, skills, and resources needed to believe that they can successfully engage in a behavior. For example, researchers successfully enhanced people’s intention to perform breast self-examination by asking them to read statements emphasizing their capability to perform [44]. As for maladaptive response reward, several longitudinal studies have demonstrated that providing information (e.g., stressing unpleasant outcomes) can be one of the successful interventions in this regard for health promotion, such as reducing unhealthy sunbathing [45]. For COVID-19 vaccination promotion, the unpleasant outcomes associated with delayed or no vaccination may be vividly shown as cases or stories as a maladaptive response reward intervention. Our findings also indicated that an enhancement of the response efficacy might encourage COVID-19 vaccination. Past studies have shown that messages informing people that an adaptive behavior was effective (vs. not mentioning the effectiveness) encouraged adaptive behaviors [44]. In a review, Jarrett et al. [10] argued that dialogue-based interventions, involving social mobilization or social media, for vaccination were promising for increasing vaccination intentions and behaviors because they can effectively counter the misconceptions of vaccines and distrust of the policymakers. Last but not least, based on the varying strength of PMT constructs for high- and low-efficacy vaccines, promotion strategies toward different groups of target populations, such as in countries that adopt vaccines with relatively lower efficacy, may need to be tailored because of diverse beliefs about vaccine efficacy [46].

Our study has several limitations. First, the data were cross-sectional and non-experimental, which did not allow for causal attribution. Second, the current study did not collect information on actual COVID-19 vaccination behavior because the only measurable behavior at the time of data collection was vaccine reservation behavior, which may be misleading due to daily quota restrictions during the early stage of vaccine rollout. Although previous studies have consistently endorsed intention as a good predictor of vaccination behaviors, e.g., [47], there is a gap between vaccination intentions and vaccination behaviors. Subsequent studies are recommended to investigate the roles of the PMT factors in promoting vaccination behaviors for comparison. Third, even though we did not mention the names of particular vaccines, we could not rule out the possibility that respondents might map the high- and low-efficacy vaccines to a specific vaccine they knew—these perceptions may have influenced their evaluations of efficacy. Fourth, we did not directly assess perceived vaccine safety [37], one major determinant of vaccine intentions and behaviors, which may explain the overall relatively low rates of positive vaccine intentions, even for vaccines that have a 95% efficacy.

## 5. Conclusions

To our best knowledge, the present study is the first study using a probability community sample to investigate the relation between PMT factors and COVID-19 vaccination intentions. Our findings supported that, within the PMT framework, cognitive factors related to both threat appraisal and coping appraisal processes were associated with vaccination intentions. Aside from lending empirical support to the applicability of PMT to COVID-19 vaccination intentions, the present study also extended the PMT framework to studying COVID-19 vaccination intentions under different scenarios of vaccine efficacy. Given the sensitivity of PMT factors to different groups of people across scenarios, the findings indicated the necessity to address inconsistent findings on vaccination intentions due to variations in the vaccines and to devise tailored enhancement strategies based on PMT factors for promoting intentions for receiving vaccines of different efficacy levels.

## Figures and Tables

**Table 1:** The effect of demographic variables on vaccination intentions.

Variables	Categories	Percentage	Mean (*SD*) of Vaccination Intention			Low- vs. High- Efficacy (*t*-Test)
			General	Low-Efficacy	High-Efficacy	
1. Sex	Male	49.2%	3.06 (0.99)	2.97 (1.02)	3.43 (1.12)	−8.04 ***
	Female	50.8%	3.06 (1.05)	3.03 (1.06)	3.66 (1.11)	−10.39 ***
*Comparing men with women: t* (*df = 462*)			−0.08	−0.58	−2.25 *	--
2. Age (years)	18–24	8.4%	3.34 (1.12)	3.13 (1.10)	4.00 (1.07)	−6.12 ***
	25–34	30.8%	2.91 (0.90)	2.95 (0.93)	3.35 (1.02)	−5.82 ***
	35–44	32.6%	2.95 (0.89)	2.90 (0.90)	3.23 (1.02)	−5.13 ***
	45–54	12.0%	3.17 (1.03)	3.17 (1.14)	3.81 (1.16)	−5.19 ***
	55–64	7.8%	3.53 (1.08)	3.47 (1.05)	4.28 (0.96)	−5.85 ***
	65 and above	8.4%	3.26 (1.22)	3.11 (1.30)	3.94 (1.26)	−4.17 ***
*Comparing the six age groups: F* (*df = 5, 445*)			3.35 **	2.22	9.73 ***	--
3. Education	Primary or lower	5.8%	2.84 (1.31)	2.60 (1.19)	3.68 (1.44)	−4.69 ***
	Secondary school	34.9%	3.20 (0.93)	3.17 (0.98)	3.68 (1.11)	−6.88 ***
	College or higher	59.3%	3.02 (1.00)	2.96 (1.02)	3.45 (1.08)	−10.26 ***
*Comparing the three educational levels: F (df = 2, 463)*			1.44	4.39 *	2.32	--

Note. * *p* < 0.05; ** *p* < 0.01; *** *p* < 0.001.

**Table 2:** Descriptive statistics and bivariate correlations.

	1	2	3	4	5	6	7	8	9
1. General Vaccination Intention	1								
2. Low-efficacy Vaccine Intention	0.75 ***	1							
3. High-efficacy Vaccine Intention	0.70 ***	0.67 ***	1						
4. Perceived Severity	0.07	0.06	0.30 ***	1					
5. Perceived Vulnerability	−0.09	0.06	−0.01	0.09	1				
6. Maladaptive Response Reward	−0.45 ***	−0.36 ***	−0.46 ***	−0.24 ***	0.03	1			
7. Self-efficacy	0.42 ***	0.32 ***	0.34 ***	0.10 *	−0.05	−0.27 ***	1		
8. Response Efficacy	0.43 ***	0.37 ***	0.27 ***	0.04	−0.02	−0.25 ***	−0.43 ***	1	
9. Response Cost	−0.34 ***	−0.20 ***	−0.39 ***	−0.27 ***	0.08	0.61 ***	−0.21 ***	−0.14 **	1
* M*	3.06	3.02	3.52	4.06	2.54	3.11	3.73	3.77	2.84
* SD*	1.00	1.02	1.10	0.63	0.84	0.89	0.93	0.81	0.83

Note. * p < 0.05; ** *p* < 0.01; *** *p* < 0.001.

**Table 3:** Hierarchical regressions of intentions toward COVID-19 vaccination.

Predictor	General Vaccination Intention				Low-Efficacy Vaccine Intention				High-efficacy Vaccine Intention			
	*β*	*B*	[95% CI]	*t*	*β*	*B*	[95% CI]	t	*β*	*B*	[95% CI]	*t*
**Step 1**												
(Constant)		2.85	[2.10, 3.59]	7.53 ***		2.69	[1.95, 3.44]	7.07 ***		3.04	[2.23, 3.85]	7.35 ***
Sex	0.03	0.06	[−0.13,0.25]	0.60	0.07	0.13	[−0.06, 0.32]	1.37	0.11	0.24	[0.03, 0.44]	2.26 *
Age	0.09	0.006	[−0.002, 0.02]	1.50	0.08	0.006	[−0.002, 0.02]	1.45	0.13	0.01	[0.001, 0.02]	2.21 *
Education attainment	−0.03	−0.03	[−0.12, 0.06]	−0.56	−0.03	−0.02	[−0.11, 0.07]	−0.44	−0.06	−0.06	[−0.15, 0.04]	−1.13
	Δ*R^2^* = 0.01, *p* > 0.05				Δ*R^2^* = 0.02, *p* > 0.05				Δ*R^2^* = 0.04, *p* < 0.001			
**Step 2**												
(Constant)		3.34	[2.26, 4.42]	6.08 ***		2.18	[1.01, 3.35]	3.66 ***		2.83	[1.61, 4.05]	4.56 ***
Sex	0.00	−0.01	[−0.17, 0.15]	−0.10	0.06	0.12	[−0.06, 0.29]	1.31	0.04	0.09	[−0.09, 0.27]	0.96
Age	−0.01	0.00	[−0.01, 0.01]	−0.14	−0.01	0.00	[−0.01, 0.01]	−0.12	0.00	0.00	[−0.01, 0.01]	0.04
Education attainment	−0.06	−0.05	[−0.13, 0.02]	−1.33	−0.03	−0.02	[−0.11, 0.06]	−0.59	−0.09	−0.08	[−0.17, 0.00]	−1.91
Perceived severity	−0.08	−0.12	[−0.25, 0.01]	−1.77	−0.03	−0.05	[−0.20, 0.09]	−0.71	0.17	0.30	[0.15, 0.45]	3.88 ***
Perceived vulnerability	−0.07	−0.08	[−0.18, 0.02]	−1.59	0.08	0.09	[−0.01, 0.20]	1.75	0.00	−0.01	[−0.11, 0.11]	−0.09
Maladaptive response reward	−0.28	−0.30	[−0.42, −0.19]	−5.37 ***	−0.30	−0.33	[−0.45, −0.21]	−5.21 ***	−0.25	−0.31	[−0.43, −0.18]	−4.81 ***
Self-efficacy	0.22	0.24	[0.14, 0.33]	4.93 ***	0.15	0.16	[0.06, 0.26]	3.15 ***	0.19	0.22	[0.12, 0.33]	4.18 ***
Response efficacy	0.23	0.29	[0.17, 0.40]	5.01 ***	0.22	0.28	[0.16, 0.40]	4.48 ***	0.07	0.10	[−0.03, 0.23]	1.54
Response cost	−0.10	−0.12	[−0.24, 0.01]	−1.88	0.05	0.06	[−0.08, 0.19]	0.84	−0.13	−0.17	[−0.31, −0.04]	−2.47 *
	Δ*R^2^* = 0.32, *p* < 0.001				Δ*R^2^* = 0.21, *p* < 0.001				Δ*R^2^* = 0.27, *p* < 0.001			

* p < 0.05; *** *p* < 0.001.

## Data Availability

The data presented in this study are available on reasonable request.

## Appendix A

**Table A1:** The COVID-19 Vaccination Intentions Survey.

1.	COVID-19 Related Experiences
	Have you or your family members or friends been diagnosed with COVID-19, had close contact with confirmed COVID-19 patients, or been quarantined because of the COVID-19 pandemic? [Please select all applicable options]
	□ None □ I have been diagnosed with COVID-19 □ I have had close contact with confirmed COVID-19 patient(s) □ I have been quarantined due to the pandemic □ My family member(s) have been diagnosed with COVID-19 □ My family member(s) have had close contact with confirmed COVID-19 patient(s) □ My family member(s) have been quarantined due to the pandemic □ My friend(s) have been diagnosed with COVID-19 □ My friend(s) have had close contact with confirmed COVID-19 patient(s) □ My friend(s) have been quarantined due to the pandemic
**2.**	**COVID-19 Vaccination Intention**
	In the next six months, to what extent are you willing to receive COVID-19 vaccines in the following scenarios? Please use 1–5 points to rate, in which 1 = very low, 2 = low, 3 = medium, 4 = high, 5 = very high.
	(a) If COVID-19 vaccines are available locally, to what extent are you willing to get vaccinated? (b) If COVID-19 vaccines are available locally and you are told that it will protect 50% of those who received the vaccines*, to what extent are you willing to get vaccinated? (* according to controlled clinical trials) (c) If COVID-19 vaccines are available locally and you are told that it will protect 95% of those who received the vaccines*, to what extent are you willing to get vaccinated? (* according to controlled clinical trials)
**3.**	**Protection Motivation Theory (PMT) variables**
	To what extent do you agree with the following statements about COVID-19 and COVID-19 vaccines? Please use 1–5 points to rate, in which 1 = strongly disagree, 2 = disagree, 3 = neither disagree nor agree, 4 = agree, 5 = strongly agree.
	***3.1 Perceived severity*** (a) If you have COVID-19, your body functions will be severely damaged, with even a possibility of death. (b) If you have COVID-19, your study or career will be harmed. (c) If you have COVID-19, you will be stigmatized, and the stigma would hurt your relationship with others.
	***3.2 Perceived vulnerability*** (a) You feel like there is a high chance for you to have COVID-19. (b) You are worried that you will contract COVID-19. (c) People around your age are at high risk of contracting COVID-19.
	***3.3 Maladaptive response reward*** (a) Not receiving COVID-19 vaccines fits your pursuit of a natural lifestyle. (b) You can avoid being a guinea pig by not receiving COVID-19 vaccines. (c) Not receiving COVID-19 vaccines can save you from troubles.
	***3.4 Response efficacy*** (a) Receiving COVID-19 vaccines can lower your risk of contracting COVID-19. (b) Receiving COVID-19 vaccines can be an effective way for you to prevent COVID-19.
	***3.5 Self-efficacy*** (a) You believe that you are able (e.g., having the time and resources) to receive COVID-19 vaccines.
	***3.6 Response cost*** (a) You may experience side effects due to COVID-19 vaccination. (b) To you, receiving COVID-19 vaccines is a waste of time. (c) To you, receiving COVID-19 vaccines is a waste of resources.
